# Icariin Ameliorates Neuropathological Changes, TGF-β1 Accumulation and Behavioral Deficits in a Mouse Model of Cerebral Amyloidosis

**DOI:** 10.1371/journal.pone.0104616

**Published:** 2014-08-07

**Authors:** Zhi-Yuan Zhang, Chaoyun Li, Caroline Zug, Hermann J. Schluesener

**Affiliations:** Division of Immunopathology of the Nervous System, Institute of Pathology and Neuropathology, University of Tuebingen, Tuebingen, Germany; University of S. Florida College of Medicine, United States of America

## Abstract

Icariin, a major constituent of flavonoids from the Chinese medicinal herb Epimedium brevicornum, exhibits multiple biological properties, including anti-inflammatory, neuroregulatory and neuroprotective activities. Therefore, Icariin might be applied in treatment of neurodegenerative disorders, including Alzheimer's disease (AD), which is neuropathologically characterized by β-amyloid aggregation, hyperphosphorylated tau and neuroinflammation. Potential therapeutic effects of Icariin were investigated in an animal model of cerebral amyloidosis for AD, transgenic APP/PS1 mouse. Icariin was suspended in carboxymethylcellulose and given orally to APP/PS1 mice. Therapeutic effects were monitored by behavioral tests, namely nesting assay, before and during the experimental treatment. Following an oral treatment of 10 days, Icariin significantly attenuated Aβ deposition, microglial activation and TGF-β1 immunoreactivity at amyloid plaques in cortex and hippocampus of transgenic mice 5 months of age, and restored impaired nesting ability. Our results suggest that Icariin might be considered a promising therapeutic option for human AD.

## Introduction

**Figure 1:**
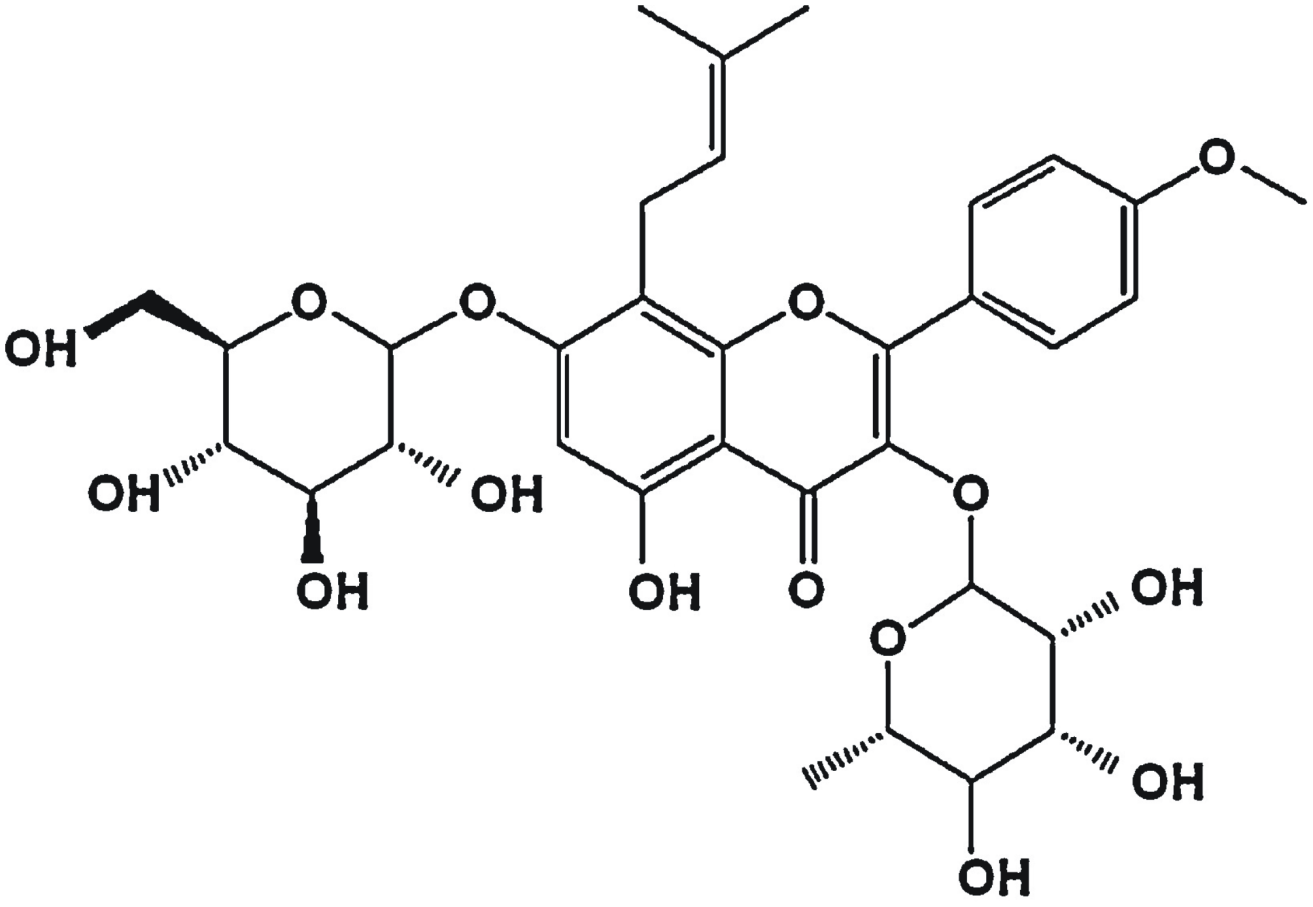
Molecular structure of Icariin.

Icariin is a natural flavonoid extracted from the Chinese tonic herb Epimedium and is considered the major pharmacologically active compound. Multi-functional Icariin possesses anti-tumor, anti-oxidant, vasorelaxant, anti-bacterial and anti-inflammatory activities [1] (Figure 1). Previous studies reported that Icariin ameliorated brain dysfunction induced by LPS [2], inhibited corticosterone-induced apoptosis in neurons [3], attenuated the ischemia/reperfusion damage to neurons [4], stimulated neurite growth [5] and thereby showed anti-neuroinflammatory and neuroprotective activities. Further, Icariin shows antidepressant-like activity [6]; protects against aluminium-induced learning and memory deficits due to its antioxidant activities; decreases Aβ1-40 content in the hippocampus of aluminium-intoxicated rodents [7]; and its metabolite Icaritin has neuroprotective effects on β amyloid-induced neurotoxicity to neuronal cells [8]. Therefore, Icariin is considered as a potential therapy against neurodegenerative diseases such as Alzheimer's disease (AD).

AD is the most common form of neurodegeneration and the major cause of dementia. AD is clinically defined by distinct behavioral and cognitive deficits, and the most characteristic neuropathological feature is extracellular deposit of aggregated β-amyloid (Aβ) peptide (amyloid plaques). It is proposed that Aβ peptides are toxic and causative in AD, contributing to memory loss, behavioral impairment and neurodegenerative pathology [9]. Beside the well known Aβ aggregation, neuroinflammation also plays an important role in the pathophysiology of this multifactorial disorder [10]. Neuroinflammation is characterized by release of numerous inflammatory mediators, microglial and astroglial activation, in particular around senile plaques [11], [12]. Neuroinflammation may contribute to neural dysfunction and cell death, establishing a self-perpetuating vicious cycle by which inflammation induces further neurodegeneration [13]. Therefore, anti-inflammatory drugs may have beneficial effects on neurodegenerative disorders, including AD [13]. In addition, up-regulated TGF-β1 has been observed in the brain of AD patients and AD animal models [14], [15]. As a potent immunomodulator and with a central role in the response to neuroinflammation, TGF-β1 is considered to be involved in pathological progression of AD.

Our aim was to study the potential therapeutic effect of Icariin in a transgenic mouse model of AD, focusing on its effect on Aβ deposition, neuroinflammation, TGF-β1 expression and behavioral deficits. We used the APP/PS1-21 double transgenic mouse model of cerebral amyloidosis, co-expressing the KM670/671NL mutated human amyloid precursor protein and the L166P mutated human presenilin 1 (APP/PS1-21 mice) under the neuron-specific Thy1 promoter element. In this transgenic line, amyloid deposition occurred as early as 2 months in the cortex and 4 months in the hippocampus, accompanied by inflammatory responses and robust impairment of cognitive function [16]–[18].

## Materials and Methods

### Animals

Male APP/PS1-21 mice with a C57BL/6J background were obtained from Prof. M. Jucker. Heterozygous male APP/PS1-21 mice were bred with wild-type C57BL/6J females (Charles River Germany, Sulzfeld Germany). Offsprings were tail snipped and genotyped using PCR. Animals were housed under a 12 h light-12 h dark cycle with free access to food and water. Mouse diets were provided by SSNIFF Spezialdiaeten GmbH (Soest, Germany), diet number V1124-703 was for breeding pairs and diet number V2534-703 was for all the other mice. All experiments and protocols were licensed and approved by regional Administrative Council (Regierungspräsidium) Tuebingen according to The German Animal Welfare Act (TierSchG) of 2006.

### Materials

Icariin (>98%) was purchased from MR Natural Product Co., Ltd. (Xi'an, China). For oral treatment, Icariin was suspended in 1% carboxymethylcellulose (CMC, Hercules-Aqualon, Düsseldorf, Germany) at a concentration of 12.5 mg/ml (Icariin/CMC solution).

### Treatment with Icariin

14 transgenic APP/PS1-21 mice 5 months of age, 6 males and 8 females, were separated into two groups: group 1 received a 10-days Icariin treatment (100 mg/kg by daily gavage); group 2, as control, received the same volume of 1% CMC dissolved in water (approx. 200 ul).

### Design and evaluation of nest construction assay

A nest construction assay [19] was modified to determine the deficits in affiliative/social behavior of APP/PS1 mice and potential changes following treatment.

Mice were individually housed for at least 24 hours in clean plastic cages with approximately 1 cm of wood chip bedding lining the floor and identification cards coded to render the experimenter blind to gender, age, and genotype of mice. Two hours prior to the onset of the dark phase of the light cycle, individual cages were supplied a 20×20 cm piece of paper towel torn into approximately 5×5 cm squared pieces. The next morning (approximately 16 hours later), cages were inspected for nest construction. Pictures were taken prior to evaluation for documentation. Paper towel nest construction was scored by a 3 point system: 1 = no biting or tears on the paper, 2 = moderate biting and/or tears on the paper but no coherent nest (not grouped into a corner of the cage) and 3 = the vast majority of paper torn into pieces and grouped into a corner of the cage [19].

### Immunohistochemistry (IHC) and image evaluation/analysis

Icariin-treated and control mice were sacrificed after 10-days-treatment. According to our previous data, Aβ deposition starts as early as 2 months of age, but develops very fast (exponential phase) around the age of 5 months. We presumed that a potential treatment may affect Aβ deposition most effectively during the exponential phase; therefore mice at an age of 5 months were used. We balanced the treatment and control groups by gender-, age and body weight matched mice. Afterwards, we analysed possible difference in plaque burden and behavioural deficits between genders within a group and found no significant differences. But the brain sizes of female mice are generally smaller than that of males. Therefore, we introduced analysis of IR percentage to rule out interference of brain size differences between genders or single individuals of the same gender. Mice were deeply anesthetized with ether and perfused intracardially with 4°C, 4% paraformaldehyde in PBS. Brains were quickly removed and post-fixed in 4% paraformaldehyde overnight at 4°C. Post-fixed brains were cut into two hemispheres; hemispheres were embedded in paraffin, serially sectioned (3 µm) and mounted on silane-covered slides. Hemispheres sections were stained with HE or IHC as described previously [20]. The following antibodies were used: anti-β-amyloid (1∶100; Abcam, Cambridge, UK) for Aβ deposition, anti-Iba-1 (1∶200; Wako, Neuss, Germany) for activated microglia, anti-GFAP (1∶500; Chemicon (Millipore), Billerica, MA, US) for astrocytes and anti-transforming growth factor beta 1 (TGF-β1) (1∶50, Santa Cruz, Dallas, Texas, US). The rabbit polyclonal anti-β-amyloid antibody (ab2539) was generated against the synthetic peptide DAEFRHDSGYEVHH conjugated to KLH, corresponding to amino acids 1–14 of Human β-amyloid. Positive and negative controls were routinely performed in each staining experiment to validate the immunohistochemical staining quality and results. Negative controls were performed by deletion of primary antibody and no unspecific staining was observed.

After immunostaining, sections were examined by light microscopy (Nikon, Düsseldorf, Germany). Aβ deposition, Iba-1 and GFAP immunostaining were evaluated at cross-sections of hemispheres, especially focused on cortex and hippocampus. All sections were randomly numbered and analysed by two observers independently, who were not aware of the treatment and time points. Aβ plaques, Iba-1^+^ and GFAP^+^ cells in cortex and hippocampus were counted under a microscope with a 50-fold magnification, by clear deposition for plaques and clear counter staining of cellular nuclei for cells. Further, images of hemisphere cross-sections were captured using Nikon Cool-scope (Nikon, Düsseldorf, Germany) with fixed parameters; cortex and hippocampus of the images were outlined and analyzed using the software MetaMorph Offline 7.1 (Molecular Devices, Toronto, Canada). Area percentages of specific immunoreactivity (IR) of interesting regions were selected by color threshold segmentation and calculated. All parameters were fixed for all images of a particular staining. Results were given as arithmetic means of plaque/cell counts or area percentages of IR to interest areas on cross-sections and standard errors of means (SEM).

### Statistical analysis

Difference of plaque/cell counts, area percentages of staining and scores of nest construction between treatments and controls were analysed by unpaired t-tests (Graph Pad Prism 5.0 software). For all statistical analyses, significance levels were set at P<0.05.

## Results

### Effect of Icariin treatment on behaviour impairments

Effect of Icariin treatment was monitored using a harmless behavioural test, the nesting assay. As a born instinct, nesting behaviour is important for small rodents in heat conservation, reproduction and shelter. As a baseline of this assay, an impaired nesting ability of these transgenic mice was confirmed in our previous study, compared to age- and gender matched naïve mice [21]. Nest construction with paper towel material was explored using a 3 point scaling system.

**Figure 2:**
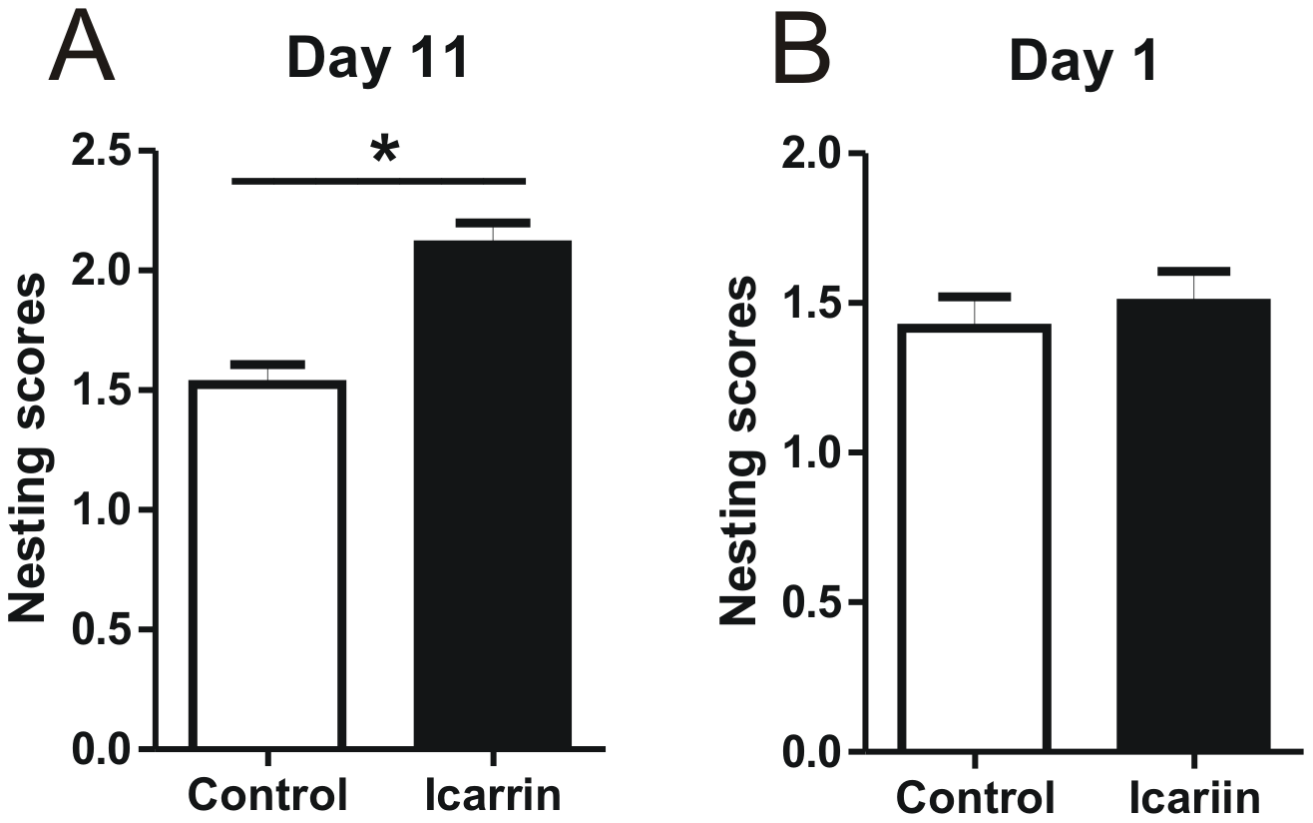
Effect of Icariin on impaired nesting ability. Therapeutic effects of Icariin were monitored using a nesting assay before and during the experimental treatment. Nest construction was explored with paper towel material using a 3 point scaling system in APP/PS1 mice. A: A significant difference between treatment and control group was observed after a 10-days treatment, namely at Day 11. B: No significant difference between the Icariin treatment and the control group could be observed right at the beginning of treatment, namely at Day 1.

A significant difference between treatment and control groups of these age- and gender-matched APP/PS1 mice was already observed at Day 11, after 10 days of treatment (control = 1.5±0.1, Icariin = 2.1±0.2, p<0.05, n = 6) (Figure 2A). In the Icariin treatment group, relatively immediate chewing and tearing of the paper towels were observed; paper towels were torn into pieces and grouped into a corner of the cage. In contrast, transgenic mice from the control group investigated and slightly chew but did not really destruct the paper towels; paper towels were found all over in the cage, not grouped or not in the corners. As a control, no significant difference between treatment and control groups could be observed right at the beginning of treatment, namely at Day 1 (control = 1.4±0.1, Icariin = 1.5±0.2, p>0.5, n = 6) (Figure 2B).

After acquisition of these positive results, all mice were sacrificed and the effects of Icariin on neuropathological changes were further investigated.

### Effects of Icariin on amyloid plaques

**Figure 3:**
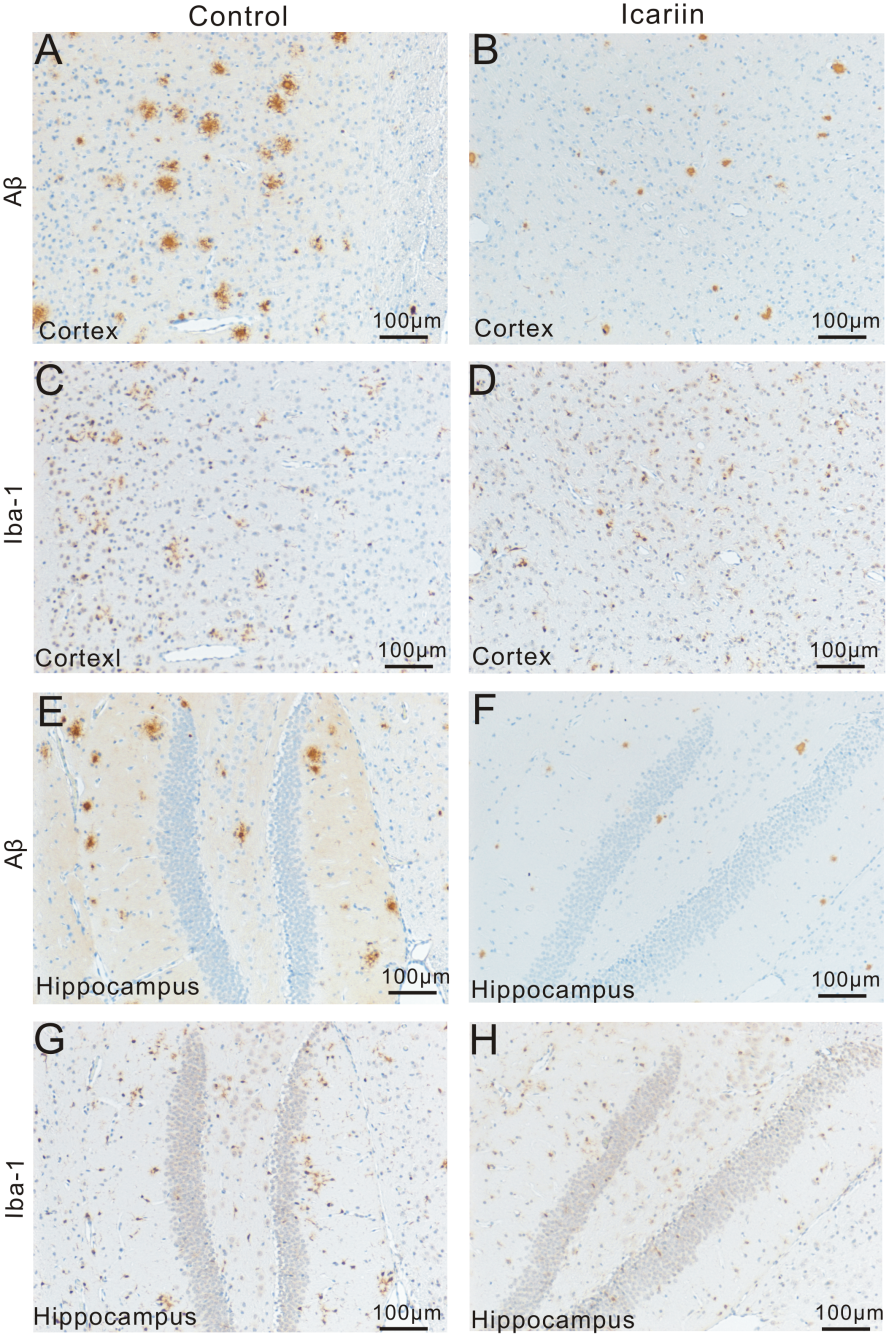
Therapeutic effect of Icariin on Aβ deposition and microglial activation. Representative microimages show the changes in Aβ deposition and microglial activation in cortex and hippocampus following Icariin treatment. A–B and E–F: In both cortex (A–B) and hippocampus (E–F) of APP/PS1 mice from the Icariin group (B and F), reduced numbers of Aβ plaques with relatively smaller size were observed, compared to the control group (A and E). C–D (cortex) and G–H (hippocampus): According to serial sections of Aβ staining, most Iba-1^+^ microglia accumulated at or surrounding Aβ plaques. In the treatment group (D and H) fewer numbers of Iba-1^+^ cells and smaller IR area of Iba-1 could be seen, compared to the control group (C and G).

In brain of APP/PS1 transgenic mice from the control group, Aβ plaques were distributed throughout the whole cortex. Plaques were of different sizes, most small plaques had dense cores and larger plaques mainly consisted of a dense core surrounded by a large halo of diffuse amyloid (Figure 3A). In the hippocampus, plaque density was lower and most plaques were of smaller size (Figure 3E).

**Figure 4:**
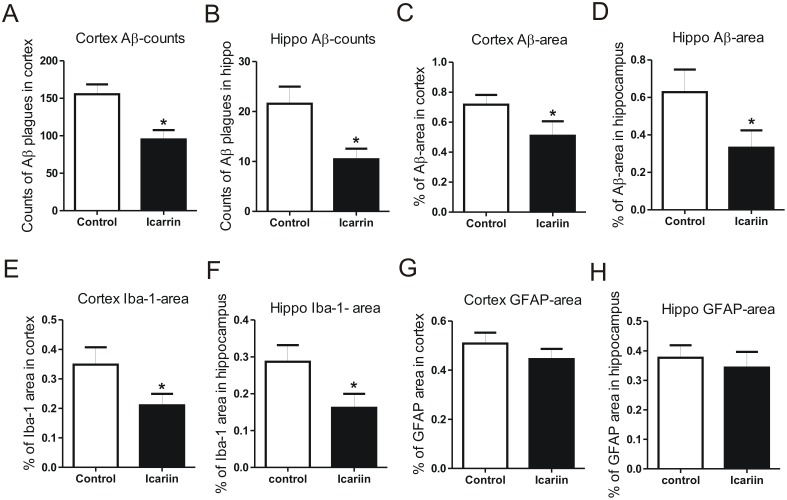
Icariin reduced β-amyloid (Aβ) deposition and microglial activation. Differences of Aβ plaque counts and Aβ^+^/Iba-1^+^/GFAP^+^ area percentages between treatment and control were analysed by unpaired t-test and results are represented in the bar graphs. A and B: In cortex and hippocampus of transgenic mouse brains from the Icariin group, numbers of amyloid plaques were significantly reduced. C and D: IR area percentages of Aβ staining were highly significantly reduced. E and F: Following Icariin treatment, Iba-1 IR was significantly reduced in cortex and hippocampus. G and H: GFAP IR was not significantly changed by Icariin treatment.

The transgenic mice received Icariin suspension or vehicle by gavage for 10 days. The treatment with Icariin attenuated neuropathological change, compared to the age- and gender-matched control mice. The Icariin treatment reduced the plaque counts significantly in cortex (control = 155.4±13.2, Icariin = 95.0±12.6, p<0.05, n = 6) and hippocampus (control = 21.6±3.4, Icariin = 10.4±2.1, p<0.05; n = 6) (Figure 4A and B). Further analysis of the micro photos showed highly significantly decreased Aβ IR area in both cortex and hippocampus from the Icariin treatment group (cortex: control = 0.72±0.06%, Icariin = 0.51±0.10%, p<0.05; hippocampus: control = 0.63±0.12%, Icariin = 0.33±0.09%, p<0.05; n = 6) (Figure 4C and D). Notably, Aβ plaques had a smaller size and fewer branches (Figure 3A and B).

### Effects of Icariin on microglial activation

**Figure 5:**
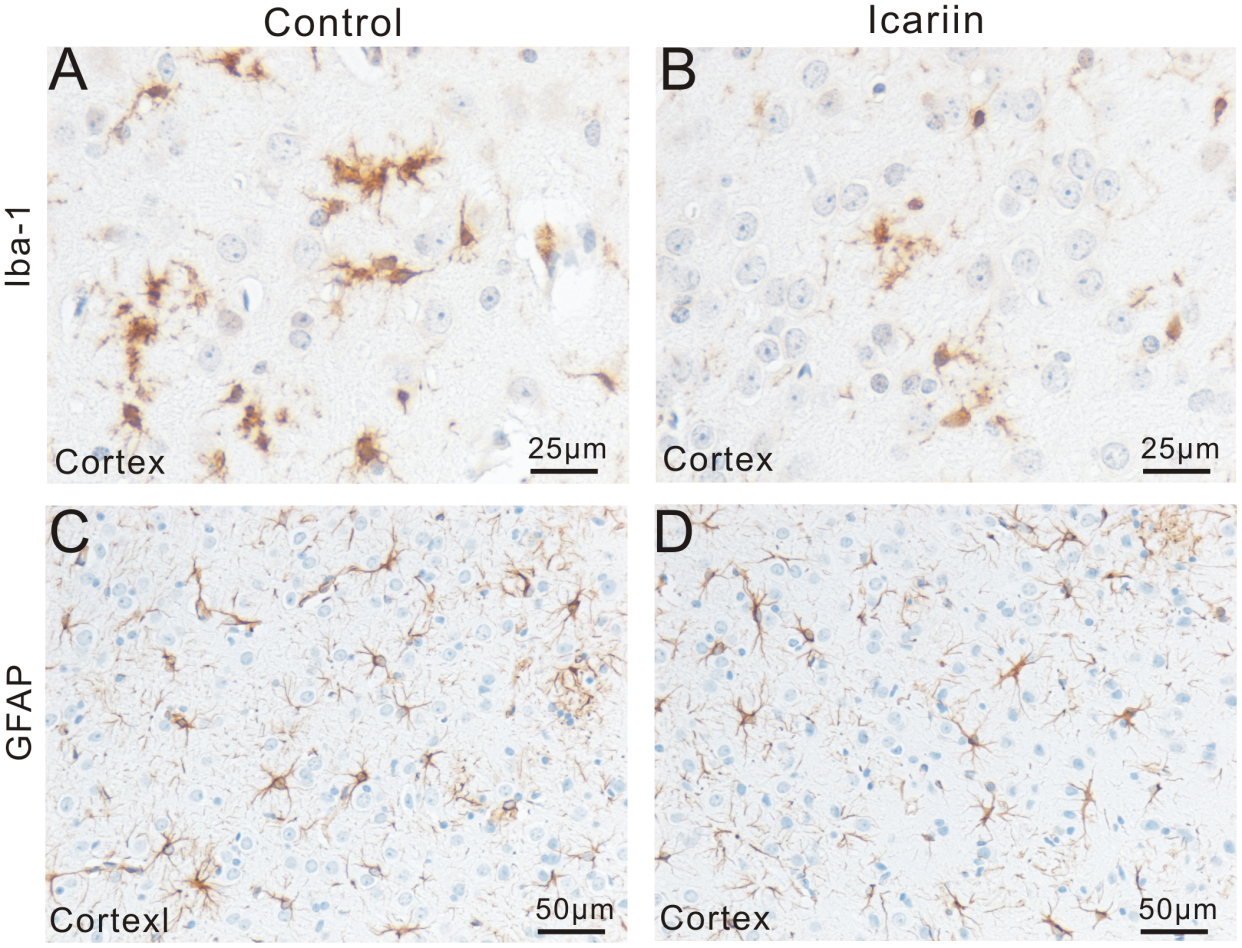
Therapeutic effect of Icariin on microglial and astrocytic activation. A and B: Representative microimages with higher magnification show the changes in microglial activation in cortex following Icariin treatment. C and D: Representative microimages show typical morphology of astrocytes, including stellate shape and multiple branched processes. No massive aggregation of GFAP^+^ cells and no obvious changes of GFAP IR were observed.

In both cortex and hippocampus of non-transgenic mice, Iba-1 staining could be barely observed. In transgenic mice, however, amoeboid Iba1^+^ microglia were observed clustered around amyloid deposits in both control and treatment groups (Figure 3C and G, Figure 5A with higher magnification), according to comparison with Aβ staining of serial sections. Numbers of Iba-1^+^ cells in Icariin treatment group were significantly less than those of the control group. Notably, in the cortex; decreased Iba-1^+^ cells were also less clustered around plaques (Figure 3D and H, Figure 5B with higher magnification). Further analysis showed that Icariin treatment significantly reduced the IR area of Iba-1 in cortex (control = 0.35±0.06%, Icariin = 0.21±0.04%, p<0.05, n = 6), and hippocampus (control = 0.29±0.03%, Icariin = 0.16±0.05%, p<0.05, n = 6) (Figure 4E and F). These results indicate reduced microglial activation.

### Effects if Icariin on astrocyte GFAP expression

Numerous GFAP^+^ cells (GFAP IR) were widely distributed throughout the hippocampus and cortex. They all showed typical morphology of astrocytes, including stellate shape and multiple branched processes; some of them had the typical morphology of perivascular astrocytes (Figure 5C and D). Double staining showed that all Aβ plaques were surrounded or covered by GFAP IR, but no massive aggregation of GFAP^+^ cells was related to Aβ plaques. Further analysis showed that no significant changes in GFAP IR were observed between Icariin treatment and controls; GFAP-IR area was only slightly decreased by Icariin treatment: in cortex (control = 0.51±0.05%, Icariin = 0.45±0.04%, p>0.05, n = 6), and in hippocampus (control = 0.38±0.04%, Icariin = 0.34±0.05%, p>0.05, n = 6) (Figure 4G and H).

### Expression pattern of TGF-β1 and effect of Icariin treatment on TGF-β1 IR

**Figure 6:**
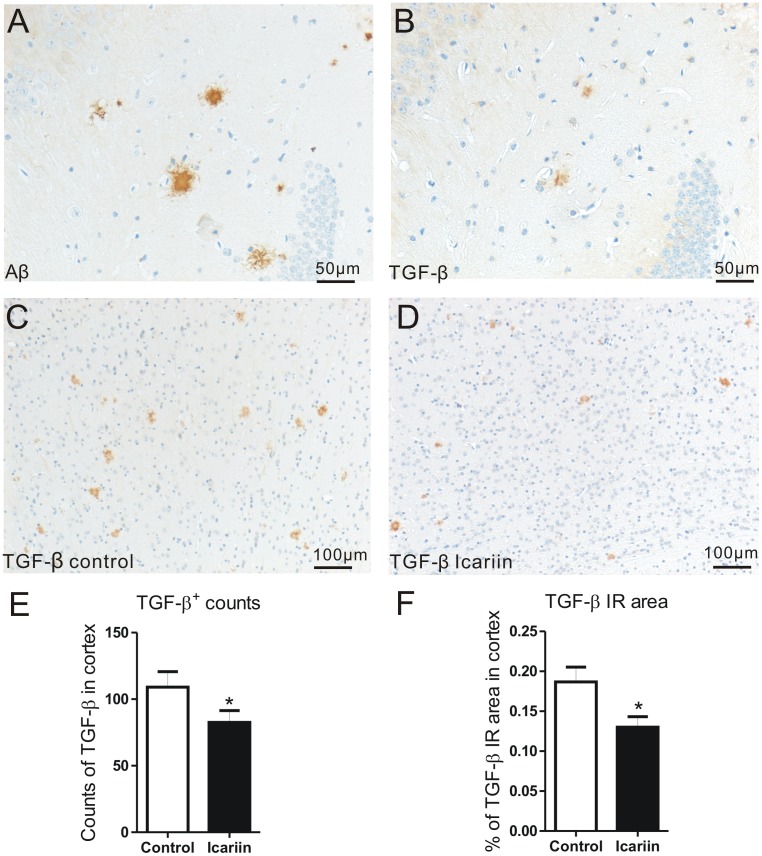
Effect of Icariin on TGF-β IR. At age of 5 months, increased TGF-β1 IR was observed in cortex of transgenic mice. A and B: In comparison of serial section of Aβ staining (A), TGF-β1 IR (B) had generally less intensity and smaller IR area, and was mainly located at or around Aβ plaques, but could be barely seen on glial cells or neurons. C and D: Much less intensity and smaller TGF-β1 IR area were seen in the Icariin treated group (D), compared to the control group (C). E and F: Statistical evaluation showed significant reductions in counts of TGF-β1^+^ plaques and area of TGF-β1 IR.

Until 3 months of age, TGF-β1 IR could not be seen in cortex or hippocampus of transgenic and naïve mice (data not shown). At age of 5 months, increased TGF-β1 IR was observed and mainly located on or around Aβ plaques, but could barely be seen on glial cells or neurons, according to the results of double staining and in comparison with serial sections of Aβ staining (Figure 6A and B). TGF-β1 IR therefore presented an Aβ plaque-like distribution pattern, but with much less intensity and smaller IR area compared to Aβ staining, as presented in the figure 6 (Figure 6A and B). TGF-β1 IR was more concentrated at the range of plaques and diffused branches, less in the center of plaques.

Following the 10 days-treatment with Icariin, TGF-β1 IR was obviously reduced in brains of transgenic mouse, especially in cortex (Figure 6C and D for control and Icariin group respectively). Numbers of TGF-β1 stained plaques in cortex were significantly reduced (control = 109.0±11.7, Icariin = 82.6±8.8, p<0.05, n = 6) and areas of TGF-β1 IR in cortex were also significantly decreased (cortex: control = 0.19±0.02%, Icariin = 0.13±0.01%, p<0.05) (Figure 6E and F).

## Discussion

In this work we report therapeutically beneficial effect of Icariin, a flavonoid from the Chinese medicinal herb Epimedium brevicornum (Herba epimedii), in a rodent APP/PS1 model of cerebral amyloidosis for AD. A 10-days oral treatment by Icariin significantly attenuated Aβ deposition, microglial activation and TGF-β1 IR in both cortex and hippocampus of APP/PS1 mice at an age of 5 months, and restored impaired nesting behavior as well.

As a tonic herbal in traditional Chinese medicine, Epimedium brevicornum has been used to treat various kinds of disorders, such as hypertension, coronary heart disease, osteoporosis, menopause syndrome, breast lump, rheumatism, arthritis or hypogonadism [1]. The most important active constituents of this herb are prenylated, isopentenyl flavonoids. Icariin is one of these isopentenyl flavonoids, a diglycoside with a glucose group at C-7, a rhamnose group at C-3 position, and a methoxyl group at C0–4, as shown in the (Figure 1) [22], [23].

Recently, the structure-activity relationship (SAR) of flavonoids has been extensively investigated by theoretical calculations and experimental studies, including antitumor, antioxidant, vasorelaxant, antibacterial and anti-inflammatory activities [1]. As a multifunctional flavonoid, Icariin shows antioxidant [24], antidepressant [25], neuroprotective [7], anti-inflammatory activity/effects [26], [27]. Icariin also has functions in regulating bone remodeling, including enhancing osteoblastic differentiation and mineralization, inhibiting bone resorption, and inducing apoptosis of osteoclasts [28], [29]; and in controlling sexual, especially erectile dysfunction [30].

Icariin has shown efficient anti-neuroinflammatory activity against learning and memory deficits in animal models [7], through attenuating microglial activation by inhibiting NF-kappaB and p38 MAPK pathways [31]. This suggests Icariin's promising potential in aging-related neurodegenerative disease, since microglia are regarded as macrophage-like cells resident in the CNS and their activation has been implicated in many neurological disorders because of their inflammatory effects [32]. Therapeutic strategies controlling microglial activation and the excessive production of pro-inflammatory factors/molecules may be valuable to control neurodegeneration in dementia [33]. An important role of neuroinflammation involved in AD pathology has been reported from rodent models and humans [13], and attenuated neuroinflammation has been proven to contribute to reduced hallmark features of AD pathology, including Aβ-plaque accumulation [34]. Icariin significantly attenuated microglial activation in cortex of APP/PS1 transgenic mice, suggesting an inhibitory effect of Icariin on neuroinflammation, which may contribute to the ameliorated pathology and improved behaviors.

Aβ deposition in brains of transgenic mice is also significantly reduced by Icariin, following a relatively short term treatment of 10-days. It may be attributed to direct down-regulated amyloid loading [35] and attenuated neuroinflammation, because it has been reported that Icariin administrated by gavage negatively regulated beta-amyloid peptide segment 25–35 production in a rat model [35] and attenuated neuroinflammation also contributed to reduced Aβ deposition during progression of AD pathology [34].

Further, Amelioration of behavioral deficits was observed following Icariin treatment. Distinct behavioral and cognitive deficits are the most characteristic clinical feature of AD. Not only cognitive impairment but also deficits in non-cognitive/non-mnemonic behaviors are found in most AD mouse models, they are therefore considered very valuable for modeling human AD [36]. Toxic Aβ peptides and amyloid precursor protein [37], inflammatory reaction and inflammatory cytokines/molecules are directly associated with these deficits in behaviors [38]. Nesting behavior is an affiliative, social behavior and deficit in this non-mnemonic behavior is a debilitating feature of neurodegenerative diseases, including AD. A previous study reported very early (3 months) occurrence of impaired nesting ability in transgenic APP and APPPS1 mice and suggested that social deficits precede other neuropsychiatric and cognitive AD-like symptoms and can be employed as early markers of AD pathology in transgenic mouse models [39]. Cognitive impairment, however, cannot be observed in the APP/PS1 mice until 8 months of age [17].

Amelioration in impaired nesting ability was observed following our Icariin treatment. The hippocampus and the prefrontal cortex damage in mice are demonstrated to lead to reduced nesting material consumption and nest quality, indicating that the impairment of nesting behavior in these transgenic mice might be caused by toxic injury of Aβ and accompanying neuro-inflammation in related brain areas [19]. In addition to reduced amyloid accumulation and decreased neuroinflammation, Icariin can also attenuate β-amyloid-induced neurotoxicity and neurite atrophy [31], [40], all these may contribute to the improved affiliative/social behavior.

Moreover, Icariin also interferes with phosphodiesterases (PDEs), especially PDE4 and PDE5 [41], [42]. This is of interest to AD research because inhibition of PDE4 is known to reverse Aβ-induced memory deficits [43]; PDE5 inhibition is known to ameliorate synaptic sprouting [44] and axonal remodelling [45] and PDE3 inhibition is known to enhance neurogenesis [46].

TGF-β1 is a potent immunomodulator and plays a central role in the response of the brain to inflammation and injury, but the specific role of TGF-β1 in AD pathogenesis still stays elusive. Some previous studies indicated a possibly protective effect of TGF-β1 in AD by its neuroproctive function [47] and by promoting Aβ clearance through activation of microglia cells [15]. But on the other hand, up-regulated expression of TGF-β1 has been reported from brains of AD patients and AD animal models [14], [15]. Serveral studies further reported, that TGF-β1 potentiates/triggers increased production of APP and subsequent Aβ generation in murine and human astrocyte cultures [48], [49] and transgenic mice overexpressing TGF-β1 in astrocytes elicit Aβ deposition [50]. Moreover, TGF-β1 can promote vascular abnormalities and Aβ deposition in cerebral blood vessels and cerebrovascular TGF-β may contribute to inflammation in AD brains [15], [51], [52]. We observed that TGF-β1 IR in cortex of transgenic mice was reduced following Icariin treatment. This may suggest a possible mechanism of Icariin's therapeutic effect through inhibition of local TGF-β deposition, which might be in part due to the reported inhibition of TGF-β expression [53]. In our study, TGF-β1 IR was observed in or around amyloid plaques and could barely be seen on glial cells or neurons, which is in accordance with previous studies of AD brains and animal models [15], [51], [54], [55]. TGF-β1 is considered to be the most potent and ubiquitous profibrogenic cytokine and stimulates extracellular matrix (ECM) accumulation [56]. It has been reported, that Icariin protects tissue/organs from vascular pathological alterations by modulating expression of TGF-β1 [7], [57]. ECMs such as collagen are important components of Aβ plaques [58], reduced TGF-β1 IR by Icariin may therefore, at least partialy, contribute to reduction of Aβ plaques by decreasing the formation of the plaque's matrix.

Taken together, treatments with Icariin by gavage effectively ameliorated neuroinflammatory reaction and cerebral amyloidosis, reduced IR of TGF-β1 in cortex and hippocampus of transgenic APP/PS1 mice, and restored impaired nesting ability. All these results suggest that Icariin may be considered a promising therapeutic option for human AD.
